# Supplementary material for: Value of Left Ventricular Indexed Ejection Time to Characterize the Severity of Aortic Stenosis
Source: J Clin Med. 2022 Mar 28;11(7):1877. doi: 10.3390/jcm11071877 (PMC9000205; doi:10.3390/jcm11071877)
Supplement: Supplementary file 1 [file jcm-11-01877-s001.zip › jcm-1606456-supplementary.pdf]

Journal of
Clinical Medicine

MDPI

Article

Value of Left Ventricular Indexed Ejection Time to Characterize the Severity of Aortic Stenosis

Gabriele Pestelli [1,2], Valeria Pergola [3], Giuseppe Totaro [3], Marco Previtero [3], Patrizia Aruta [3], Antonella Cecchetto [3], Andrea Fiorencis [3], Chiara Palermo [3], Sabino Iliceto [3] and Donato Mele [3,*]

Supplemental Table S1. Regression Equations for Indexing LVET from Subgroups Analyses.

**Low Flow**	LVET − (2.282 x SVI) + (0.857 × HR)
**Slow Flow**	LVET − (4.125 x SVI) + (0.757 × HR)
**Normal Flow**	LVET − (1.256 x SVI) + (1.117 × HR)
**Abnormal (Low or Slow) Flow**	LVET − (3.059 x SVI) + (0.876 × HR)
**LVEF ≥50%**	LVET − (1.424 x SVI) + (1.206 × HR)
**LVEF <50%**	LVET − (1.442 x SVI) + (0.983 × HR)

The cohort used for derivation of the relative equation for indexing LVET is reported in left column. HR, heart rate; LVEF, left ventricle ejection fraction; LVET, left ventricle ejection time; SVI, stroke volume index.

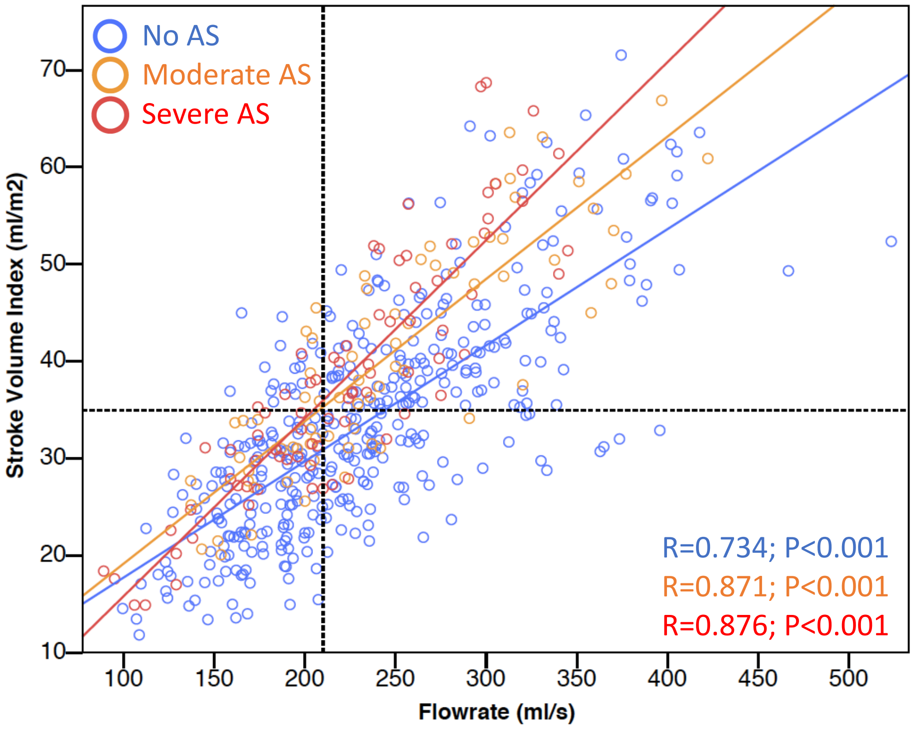

Supplemental Figure S1. Scatterplot of stroke volume index versus flowrate, according to aortic stenosis (AS) grading. Vertical dashed line indicates flowrate threshold for slow flow (210 ml/s), whereas horizontal dashed line indicates stroke volume index threshold for low flow (35 ml/m²).
